# Early-Life Stress Programs a Vulnerable Renal Phenotype Under Metabolic Challenge in a Murine Model

**DOI:** 10.3390/biom16091332

**Published:** 2026-09-14

**Authors:** Jhonatan Duque-Colorado, Bélgica Vásquez

**Affiliations:** 1 Doctoral Program in Morphological Sciences, Faculty of Medicine, Universidad de La Frontera, Temuco 4780000, Chile; jhonatan.colorado@ucaldas.edu.co; 2 Department of Basic Sciences, Faculty of Health Sciences, Universidad de Caldas, Manizales 170004, Colombia; 3 Research Group in Nutrition, Metabolism and Food Safety, Universidad de Caldas, Manizales 170004, Colombia; 4 Department of Basic Sciences, Faculty of Medicine, Universidad de La Frontera, Avenida Francisco Salazar 01145, Temuco 4811230, Chile; 5 Center of Excellence in Morphological and Surgical Studies, Universidad de La Frontera, Temuco 4811230, Chile

**Keywords:** early stress, maternal separation, high-fat diet, kidney, kidney injury, glomerular remodeling

## Abstract

Early-life stress and post-weaning exposure to a high-fat diet (HFD) have been independently associated with renal abnormalities; however, little is known about how these stressors interact to increase renal susceptibility to subsequent metabolic challenges. Therefore, this study evaluated the effects of early-life stress induced by maternal separation (MS) and post-weaning HFD on renal structure and function in male C57BL/6 mice. Animals were subjected to MS or remained unmanipulated (UM) and were subsequently fed a control diet (CD) or HFD until postnatal day 133, generating four experimental groups: UM-CD, UM-HFD, MS-CD, and MS-HFD. Renal structure was assessed by renal mass, histological analysis of the renal corpuscle, stereological quantification of glomerular numerical density (Nv) and individual glomerular volume (IGV), whereas renal function was evaluated by diuresis. MS and HFD independently increased renal mass, altered diuresis, reduced glomerular Nv, and promoted glomerular hypertrophy. Histological analysis revealed mesangial expansion, narrowing of the capsular space, focal capillary obliteration, and glomeruloparietal synechiae, which were pronounced in the MS-HFD group. Two-way ANOVA showed independent effects on Nv and a significant interaction for IGV. Reduced Nv correlated with increased IGV and diuresis. These findings indicate that early-life stress and post-weaning HFD promote complementary mechanisms of glomerular remodeling, increasing susceptibility to metabolically induced renal injury.

## 1. Introduction

Chronic kidney disease (CKD) is a major public health problem worldwide, affecting more than 10% of the population and contributing significantly to cardiovascular morbidity and mortality and healthcare costs [1]. Although CKD has traditionally been associated with risk factors acquired in adulthood, such as high blood pressure, diabetes mellitus, and obesity, growing evidence indicates that susceptibility to kidney damage can originate early through developmental programming mechanisms [2]. In this context, the Developmental Origins of Health and Disease hypothesis posits that adverse exposures during critical periods of development permanently alter organ structure and function, increasing vulnerability to chronic diseases in later life [2,3].

The kidney is particularly susceptible to this phenomenon because nephrogenesis occurs within a limited temporal window and determines the definitive nephron endowment that persists throughout life [4]. In humans, nephrogenesis ends at approximately 36 weeks of gestation, while in mice it continues during the early postnatal period [2]. In fact, approximately 51% of murine nephrons are formed between birth and the fourth postnatal day (PND), a period during which renal development remains highly active and vulnerable to early environmental aggressions [5]. Consequently, injuries occurring during this critical window can reduce nephron capacity and permanently compromise renal functional reserve.

A decrease in nephron endowment has been associated with hypertension, glomerular hypertrophy, proteinuria, and a higher risk of CKD in adulthood [6,7]. According to Brenner’s hyperfiltration hypothesis [6], nephron loss triggers compensatory hemodynamic responses in the remaining nephrons, including increased glomerular filtration per individual nephron and glomerular hypertrophy [6,8]. Although these responses initially maintain overall renal function, sustained hyperfiltration progressively promotes glomerular damage and accelerates CKD progression [9].

Early stress is a major programmer of persistent metabolic, neuroendocrine, and immunological alterations [10]. Maternal separation (MS) is one of the most widely used experimental models for studying neonatal stress to reproduce aspects of early adverse experiences through the repeated interruption of mother–infant interaction [11]. In rodents, MS activates the hypothalamic–pituitary–adrenal axis during a critical period of development, increasing exposure to glucocorticoids and altering neuroendocrine regulation [12]. In addition, MS has been shown to induce persistent inflammatory programming in the adult kidney, characterized by increased renal inflammatory markers and greater susceptibility to exacerbated immune responses [13]. Taken together, these findings suggest that neonatal stress may increase renal vulnerability to subsequent metabolic challenges.

In parallel, obesity and the consumption of a high-fat diet (HFD) have become important contributors to the development of CKD. Obesity-associated glomerulopathy is characterized by glomerulomegaly, podocyte injury, and progressive glomerulosclerosis [14]. Increasing metabolic demands require compensatory renal adaptations, including increased glomerular filtration and glomerular hypertrophy [15]. Furthermore, experimental studies have shown that post-weaning exposure to an HFD induces renal lipid accumulation, inflammation, glomerular alterations, and impaired renal function [16]. In this context, sustained hyperfiltration favors progressive glomerular remodeling and glomerulosclerosis [15].

The rationale for combining MS and HFD in the present study is grounded in the hypothesis that early-life adversity may alter the renal response to subsequent metabolic challenges. MS was selected as an experimental model of early-life stress because it occurs during a phase of active postnatal nephrogenesis in mice and can induce persistent neuroendocrine and inflammatory alterations, providing a model to investigate whether stress during renal development programs long-term renal vulnerability [2,5]. HFD was introduced after weaning to represent a later metabolic challenge capable of increasing renal hemodynamic and metabolic demands and promoting glomerular remodeling and injury. Therefore, the sequential exposure to MS followed by HFD allows investigation of whether an adverse experience during a critical developmental window modifies the renal response to a subsequent obesogenic environment [14,15,16].

Despite the growing recognition of the relationship between early adversity, obesity, and CKD risk, few experimental studies have evaluated the combined effects of neonatal stress and postnatal exposure to an HFD on glomerular morphofunctional remodeling. In particular, the integration of histological, quantitative, and functional analyses has been rarely used to jointly study early glomerular alterations associated with developmental programming, especially in models that combine neonatal stress and post-weaning exposure to an HFD.

Therefore, this study aimed to determine the effects of MS and post-weaning HFD exposure on renal structure and function in male C57BL/6 mice.

## 2. Materials and Methods

### 2.1. Animals and Experimental Design

This study used kidney tissue samples from an experimental model developed within the framework of the UFRO-DIUFRO project (code DI24-0029). In this model, researchers evaluated the effects of post-weaning MS and HFD on the structure and function of the endocrine pancreas in male C57BL/6 mice. Using the same animals allowed the analysis to be extended to other metabolically relevant tissues, avoiding the use of new animals and following the 3R principles of reduction and refinement [17]. The experimental procedures were carried out in accordance with the Guide for the Care and Use of Laboratory Animals [18] and under current animal welfare regulations. The Scientific Ethics Committee of the University of La Frontera approved the original protocol (Folio No. 067_24) on 28 May 2024. Subsequently, the Scientific Ethics Committee of the University of La Frontera evaluated and approved the specific analysis of these samples (Folio No. 140/24) on 22 January 2025. No formal study protocol was prepared or prospectively registered before the study.

To contextualize the experimental design and the conditions under which the experimental procedures produced the samples analyzed, this section describes the general characteristics of the protocol, including the origin and handling of the animals. Twenty female C57BL/6 mice were obtained from the Animal Facility of the Center of Excellence in Morphological and Surgical Studies (CEMyQ) of the University of La Frontera and underwent a 2-week acclimatization period. The animals were kept under controlled environmental conditions of temperature, humidity, and a 12:12 h light–dark cycle, with ad libitum access to water and food. For mating, mature virgin females were housed with males overnight, and mating was confirmed by observing vaginal plugs. Subsequently, the pregnant females were housed individually. Seven days before parturition, the females were evaluated twice a day (at the beginning and end of the light period) to confirm parturition. The day of birth was recorded as PND0. Litters were adjusted to seven pups each to ensure homogeneous nutrition during lactation. On PND2, the first day of MS, the sex of the pups was determined using the method described by Wolterink-Donselaar et al. [19]. This method involves visually identifying a dark spot in the anogenital region, a characteristic exclusive to newborn males with dark pigmentation. This method allows for precise and early differentiation of females, which lack this pigmentation.

### 2.2. Maternal Separation Stress

The MS stress protocol was carried out following the guidelines proposed by George et al. [20] for early weaning, thereby strengthening the validity of the early postnatal stress model, widely recognized for its ability to induce stress-associated behaviors in the early stages of development [21].

**Figure 1:**
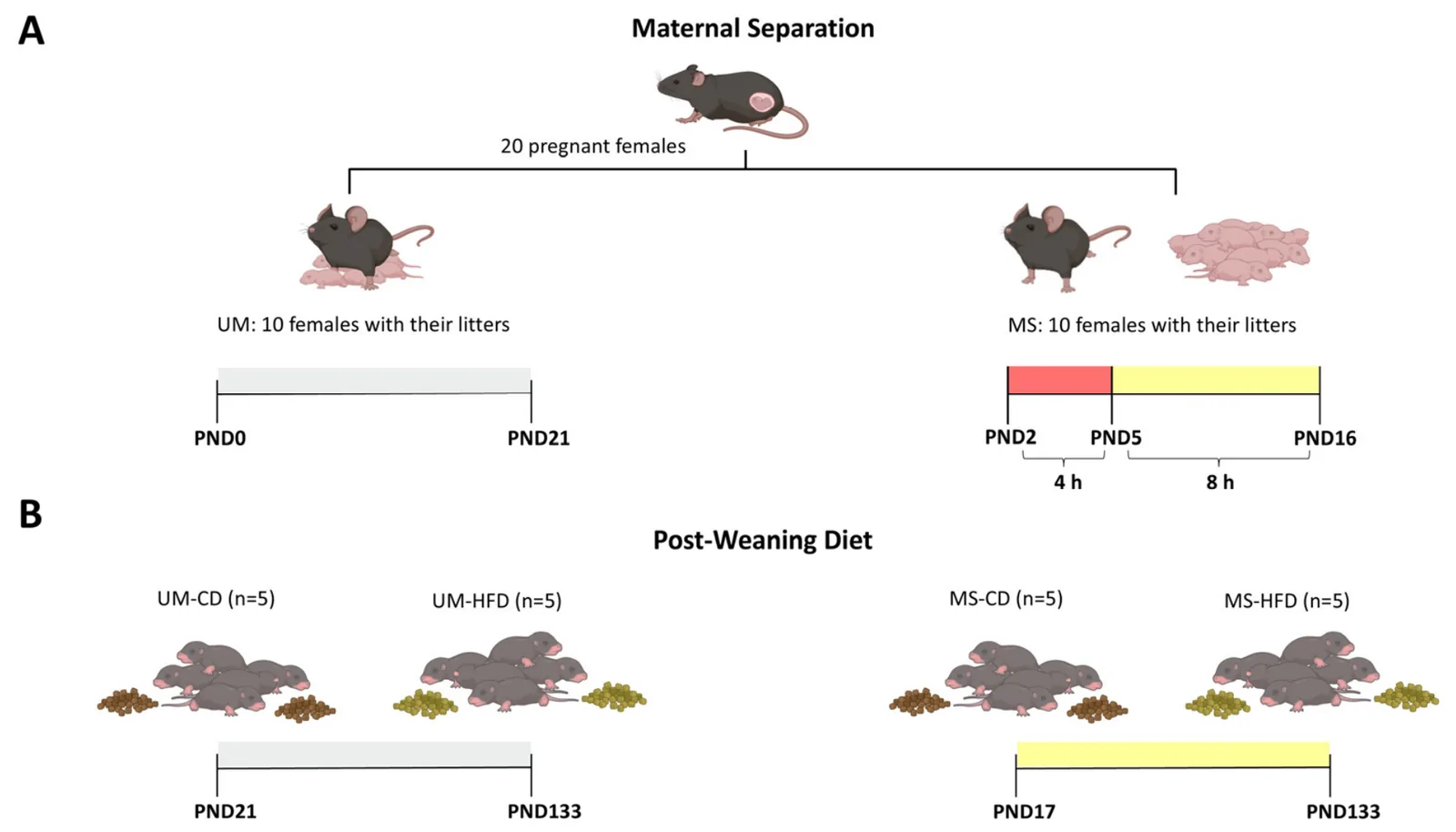
Experimental design of the study. (**A**). During the early postnatal period, male C57BL/6 mice were subjected to MS or UM. (**B**). Subsequently, from weaning until postnatal day 133, the animals were fed either a CD or HFD, establishing four experimental groups: UM-CD, UM-HFD, MS-CD, and MS-HFD. UM-CD, unmanipulated with control diet; UM-HFD, unmanipulated with high-fat diet; MS-CD, maternal separation with control diet; MS-HFD, maternal separation with high-fat diet; PND, postnatal day. Created with BioRender.com.

Thus, two experimental groups were formed: a unmanipulated (UM) group, whose offspring remained with their mothers from birth until standard weaning at postnatal day (PND) 21, and an MS group, in which the offspring were separated from their mothers during controlled periods. This model consisted of daily separations of 4 h between PND2 and PND5 and 8 h between PND6 and PND16, with early weaning at PND17 (Figure 1A). The separations were carried out between 9:00 and 13:00 h, or until 17:00 h, according to the corresponding PND. Daily inspections were carried out throughout the procedure to detect signs of dehydration or suffering, in accordance with the “Animal Monitoring” protocol [22].

During each separation session, the offspring remained in their original cage, and the mothers were moved to new cages located in an adjacent room, where they were kept under controlled environmental conditions equivalent in temperature, humidity, noise level, hygiene, and availability of food and water ad libitum. For both experimental conditions, cage cleaning was performed by a single operator, ensuring a standardized procedure and minimizing potential additional sources of stress.

### 2.3. Post-Weaning Diet

At weaning time, PND21 for the UM group and PND17 for the MS group, one male pup per litter was randomly selected to form the experimental subgroups according to the assigned diet, control diet (CD) (70 g of fat/1000 g of diet; 3.95 kcal/g) or HFD (200 g of fat/1000 g of diet; 4.95 kcal/g) (Table 1). To ensure the nutritional standardization of the study, both diets were specifically prepared for this research by PRAGSOLUÇÕES Biociências (www.pragsolucoes.com.br, Jau, SP, Brazil, accessed on 15 April 2026), containing a complete mixture of vitamins and minerals formulated according to the recommendations of the American Institute of Nutrition [23], thus ensuring the compositional consistency of the administered foods and adequate comparability between the experimental groups. Based on the above, four subgroups were formed, unmanipulated with control diet (UM-CD), unmanipulated with high-fat diet (UM-HFD), maternal separation with control diet (MS-CD), and maternal separation with high-fat diet (MS-HFD), with a total of 20 male mice included in this study (Figure 1B). The mice were fed their respective diets until PND133.

This study included only male mice, since the estrous cycle in females can introduce additional variability that affects parameters such as food intake [24]. The sample size of five animals per group was defined considering both methodological and statistical criteria widely used in experimental morphology studies. This choice also aligns with the 3R principles (replacement, reduction, and refinement) proposed by Russell and Burch [17], which aim to promote ethical and responsible use of laboratory animals. From a statistical perspective, Cruz-Orive and Weibel [25] point out that a minimum of five individuals per group provides a reasonable basis for studies of this nature. The authors indicate that, under the assumption of a uniform trend among individuals, the probability of an observed outcome occurring by chance is P = (1/2)^5^ = 0.03125, which supports the use of this sample size in this type of research.

The experimental design did not allow for the implementation of blinding procedures, since the application of the MS protocol and dietary interventions required differentiated handling of the animals according to experimental group. Furthermore, group assignments could not be kept secret during the study. Although no formal protocol was registered before the start of the investigation, the hypothesis, objectives, experimental design, and analysis strategy were established before the commencement of the study.

### 2.4. Measurement of Diuresis and Euthanasia

After PND133, the mice were individually placed in metabolic cages (41700-003, Ugo Basile, Gemonio, Italy) for 24 h before euthanasia to collect urine and quantify diuresis (mL/24 h). During this period, the animals remained in the same room as the other mice, which allowed them to maintain visual, auditory, and olfactory contact, minimizing the stress associated with isolation.

Subsequently, the animals were euthanized by an overdose of ketamine/xylazine (240/30 mg/kg), in accordance with the recommendations of the Canadian Council on Animal Care [26]. Both kidneys were carefully dissected under a stereomicroscope (Leica S6 D, Leica Microsystems, Heerbrugg, Switzerland). The left kidney was selected for analysis and was used consistently in all animals to maintain methodological uniformity. The mass of the left kidney was determined using an analytical balance (A&D Orion^®^ HR 120, A&D Technology, Saitama, Japan).

### 2.5. Histological Analysis of the Renal Corpuscle

The left kidney was fixed by immersion in 4% buffered formalin (formaldehyde 1.27 mol/L in 0.1 M phosphate buffer; pH 7.2) for 48 h at room temperature. Subsequently, the organ was dehydrated, processed using conventional histological techniques, and embedded in its entirety in Paraplast Plus (Sigma-Aldrich Co., St. Louis, MO, USA), while the right kidney was immediately snap-frozen and preserved for subsequent studies. Histological evaluation was performed on the renal corpuscles of the different experimental groups (n = 5 individual animals per group) using serial sections of 5 µm thickness stained with hematoxylin-eosin (H&E). The glomerular capsule, capsular space, glomerular capillary network, podocytes, mesangium, and cell morphology were observed, including both nuclear and cytoplasmic characteristics.

### 2.6. Stereological Analysis

**Figure 2:**
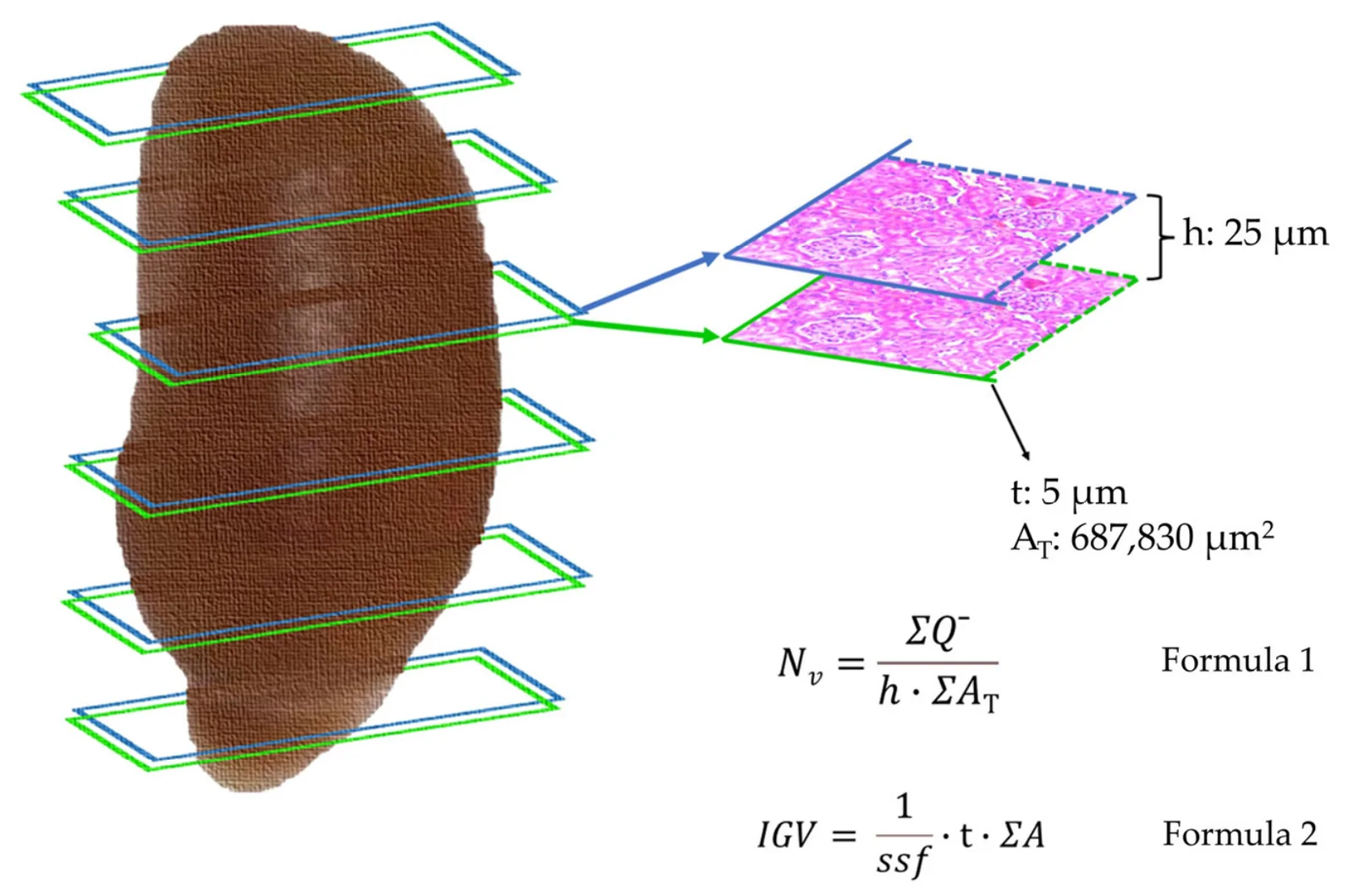
Randomized uniform systematic sampling and physical dissector for estimation of glomerular N_v_. h, height of the dissector; t, thickness of the cut; A_T_, area of the counting frame; Nv, number density; Q^−^, number of glomeruli; IGV, individual glomerular volume; ssf, sample section fraction; A, glomerular area.

Glomerular Nv was estimated using the physical dissector principle. For this, a randomized uniform systematic sampling was performed, randomly defining the starting point within the first cut plane of the renal block. Serial kidney sections 5 µm thick (t) and stained with H&E were used, selecting pairs of equidistant sections to form the dissectors, with a height (h) of 25 µm between the reference section and the search section. The physical dissector was applied bidirectionally, allowing each pair of adjacent sections to serve alternately as the reference and look-up sections. Glomerular counting was performed exclusively within the renal cortex. In each kidney, 89 counting frames, systematically distributed across the sampled cortical area, were analyzed. The area of each counting frame was 687,830 µm^2^. The sampling intensity was established according to a target coefficient of error (CE) of approximately 5%, representing the estimated sampling error associated with the stereological procedure [27]. Glomeruli were counted according to the inclusion and exclusion rules of the counting frame, with structures intersecting the permitted inclusion lines being counted and those intersecting the forbidden exclusion lines being excluded. The number of glomeruli (Q^−^) was recorded within the volume defined by the physical dissector, with Nv calculated using Formula 1, established by Sterio [27] (Figure 2). The samples were examined with a stereological microscope (Leica^®^ DM2000 LED, Leica Microsystems, Heerbrugg, Switzerland), photographed with a digital camera (Leica^®^ MC170 HD, Leica Microsystems, Heerbrugg, Switzerland) and their stereological analysis was performed using STEPanizer v1.0 software (STEPanizer, Universität Bern, Bern, Switzerland).

For IGV determination, five glomeruli were randomly selected and analyzed per mouse; however, their selection did not follow a systematic uniform random sampling scheme. As a safety margin and to ensure that the same glomerulus was monitored, 20 serial sections were obtained without losing any sections, with a thickness of 5 µm. Each profile was identified with a unique identifier (numeric labels from one to five). The IGV of the glomeruli sampled per kidney was estimated using Cavalieri’s principle [28] by means of Formula 2, where the section sampling fraction (ssf) corresponded to one (Figure 2). No correction factor for tissue shrinkage was applied; however, all renal samples were subjected to the same histological processing protocol, allowing the effects of processing-related tissue shrinkage to be considered comparable across experimental groups. The samples were examined with a stereological microscope (Leica^®^ DM2000 LED, Leica Microsystems, Heerbrugg, Switzerland), and photographed with a digital camera (Leica^®^ MC170 HD, Leica Microsystems, Heerbrugg, Switzerland), while the area of each glomerular profile (A) was determined using ImageJ v1.54g software (National Institutes of Health, Bethesda, MD, USA).

### 2.7. Statistical Analysis

Data were tested for normality (using the Shapiro–Wilk test for small samples) and homogeneity of variances (using Levene’s test) before being presented as means and standard error of the mean (SEM). Differences between groups were assessed using a two-way ANOVA and Tukey’s multiple comparisons test. Because only one male offspring per litter was included, each observation corresponded to an independent litter, which was therefore considered the experimental unit for all analyses. In all cases, a significance level of *p* < 0.05 was adopted (GraphPad Prism version 10.6.1 for Windows, GraphPad Software, Boston, MA, USA). All datasets met the assumptions for parametric analysis. Data analysis was performed using coded datasets, and group identity was revealed only after completion of the statistical analyses.

Only one male offspring per litter was included to avoid pseudoreplication, and the litter was defined as the experimental unit. No animals or experimental units were excluded after allocation, as all animals completed the MS and dietary intervention protocols and were included in the final analysis.

## 3. Results

### 3.1. Renal Mass

**Figure 3:**
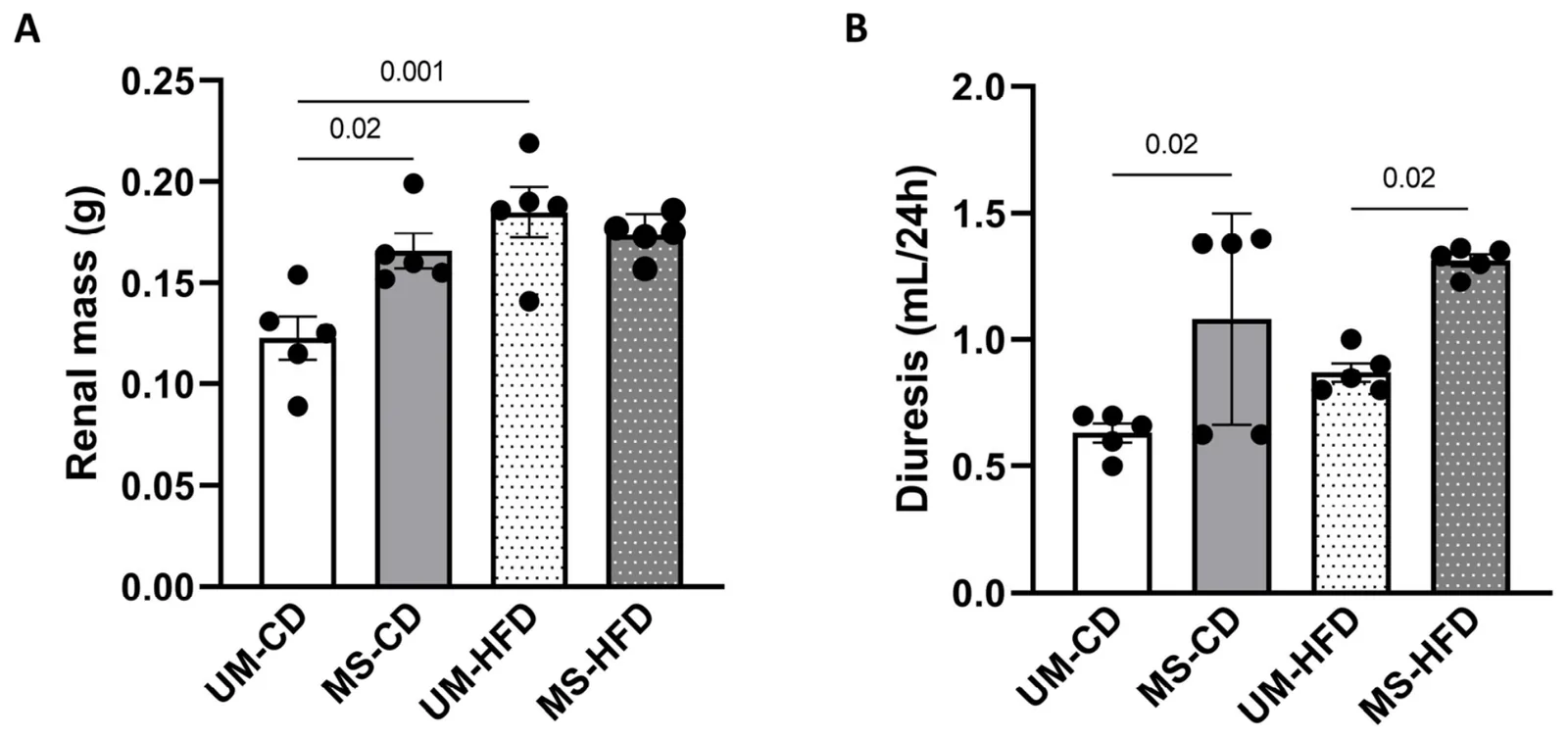
Renal changes under different experimental conditions. (**A**). Renal mass. (**B**). Diuresis. Values are presented as mean ± SEM; dots represent individual animals (n = 5 per group). Significant differences are indicated with a *p* < 0.05. UM-CD, unmanipulated with control diet; UM-HFD, unmanipulated with high-fat diet; MS-CD, maternal separation with control diet; MS-HFD, maternal separation with high-fat diet.

The UM-CD group had the lowest renal mass, representing the study baseline. In the other groups, an increase in renal mass was identified, with the highest value observed in the UM-HFD group (+51% vs. UM-CD), followed by the MS-HFD group (−6.1% vs. UM-HFD; +4.6% vs. MS-CD) and the MS-CD group (+35.2% vs. UM-CD). Multiple comparisons indicated that only the UM-CD group differed significantly from the MS-CD group (*p* = 0.02) and the UM-HFD group (*p* = 0.001). On the contrary, comparisons between groups subjected to HFD or MS did not show significant differences, with values remaining within similar ranges between UM-HFD and MS-HFD, as well as between MS-CD and MS-HFD (*p* > 0.05; Figure 3A). In this regard, it was identified that the type of manipulation did not have a significant main effect on renal mass, while diet did influence this parameter (33.1% of the total variance), with an increase in renal mass in response to HFD. Furthermore, a significant interaction was observed between the type of manipulation and diet (20.2% of the total variance, *p* = 0.01), indicating that the effect of MS on renal mass depended on the dietary condition, with MS increasing renal mass in the presence of CD and slightly decreasing it in the presence of HFD.

### 3.2. Diuresis

Diuresis showed the lowest values in the UM-CD group, representing the study baseline. Compared with this group, independent exposure to stress from MS or HFD showed moderate increases in diuresis, as evidenced by the MS-CD group (+71.2% vs. UM-CD) and the UM-HFD group (+37.7% vs. UM-CD). The highest value was observed in the MS-HFD group (+51.0% vs. UM-HFD; +21.4% vs. MS-CD), establishing an upward gradient across the experimental groups. In accordance with this behavior, the two-way ANOVA analysis revealed significant effects of both the manipulation (49.0% of the total variance) and the diet (13.5% of the total variance), consistent with the multiple comparisons analysis, in which the MS-CD group differed significantly from the UM-CD group (*p* = 0.02) and the MS-HFD group from the UM-HFD group (*p* = 0.02), while no differences were detected between the UM-CD and UM-HFD groups (*p* > 0.05). These findings indicated that, although HFD contributed to the increase in diuresis, it was not sufficient on its own to induce statistically detectable changes from baseline (Figure 3B). Along the same lines, no significant interaction was found between manipulation and diet (*p* > 0.05), suggesting that the increase in diuresis observed in the MS-HFD group would be due to a cumulative effect of both variables, rather than a synergistic action between them.

### 3.3. Histological Analysis of the Renal Corpuscle

**Figure 4:**
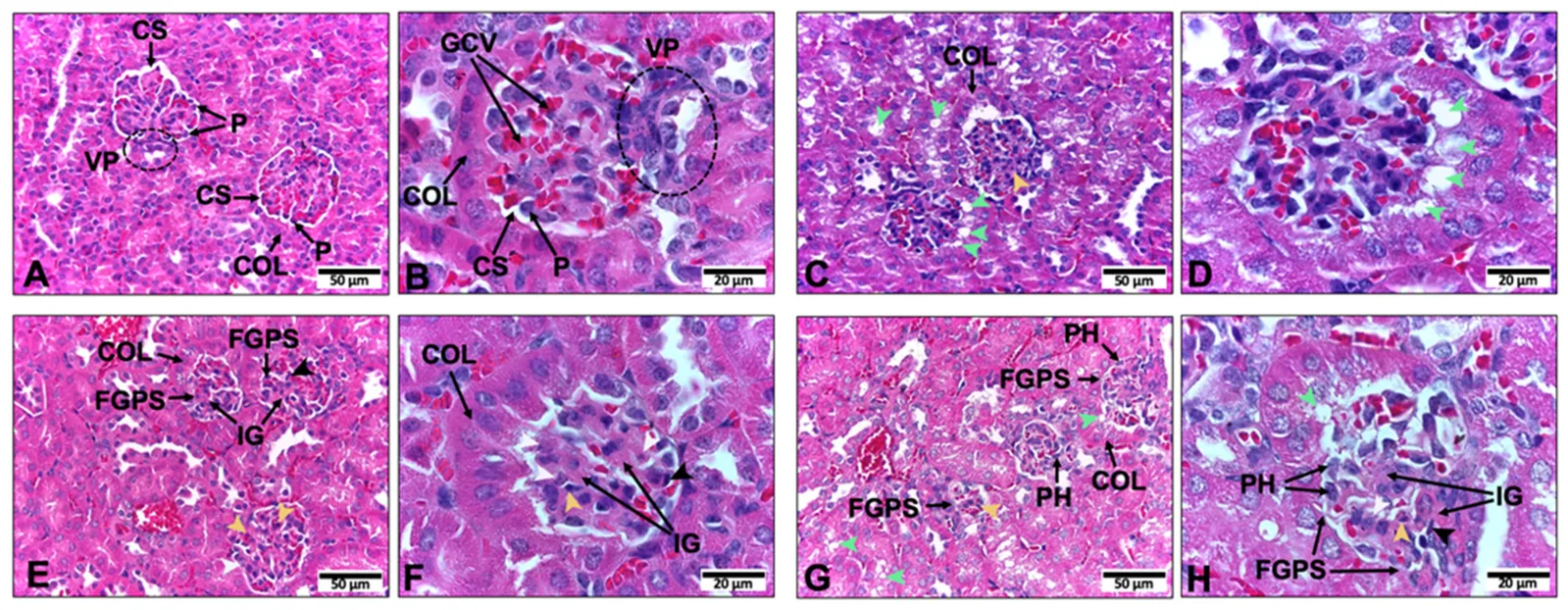
Histology of the renal corpuscle of C57BL/6 mice. (**A**,**B**). Unmanipulated with control diet. (**C**,**D**). Unmanipulated with high-fat diet. (**E**,**F**). Maternal separation with control diet. (**G**,**H**). Maternal separation with high-fat diet. VP: vascular pole; GCV: glomerular capillary vessel; COL: capsular outer layer; CS: capsular space; P: cells morphologically identified as podocytes based on their characteristic location in the visceral layer of the glomerular capsule; black arrowhead: flattened nucleus; yellow arrowhead: pyknotic nucleus; white arrowhead: karyolytic nucleus; green arrowhead: vacuolar structures morphologically compatible with lipid accumulation; IG: incipient glomerulosclerosis; FGPS: focal glomeruloparietal syneschiae; PH: cells morphologically identified as podocyte hypertrophy based on their characteristic location in the visceral layer of the glomerular capsule.

In the UM-CD group, the renal corpuscles exhibited a preserved morphology. The glomerular capsule showed a capsular outer layer lined by simple cuboidal epithelium, a characteristic described in the kidney of male C57BL/6 mice. The capsular space was clearly defined and internally delimited by the visceral layer of the glomerular capsule associated with podocytes. The glomerulus showed an adequate organization of the glomerular capillary network, supported by the mesangium distributed between the capillary loops. Furthermore, it was possible to identify the vascular poles, without evident morphological alterations (Figure 4A,B). In the UM-HFD group, the glomeruli presented a generally preserved architecture and a glomerular capillary network with a normal pattern; in addition, an increase in glomerular cellularity was identified. In some renal corpuscles, there was evidence of a greater height of the capsular outer layer, a decrease in the capsular space, and the presence of optically empty vacuolar structures morphologically compatible with lipid accumulation. The mesangial matrix remained without evident expansion, although it presented a denser arrangement (Figure 4C,D). In the MS-CD group, some renal corpuscles showed a greater height of the outer layer of the capsule. The glomeruli showed evident nuclear alterations, with the presence of pyknotic and karyolytic nuclei scattered throughout the glomerulus. Also, flattening of the nuclei of the glomerular cells was observed, accompanied by a marked expansion of the mesangial matrix, compatible with early glomerulosclerotic alterations. In this context, some glomeruli showed segmental obliteration of glomerular capillaries, as well as a greater reduction in the capsular space and the presence of focal glomerulo-parietal synechiae (Figure 4E,F). Regarding the MS-HFD group, the glomeruli showed more pronounced nuclear alterations, with abundant pyknotic and karyolytic nuclei distributed throughout the glomerulus, as well as the presence of optically empty vacuolar structures morphologically compatible with lipid accumulation. At the cellular level, an increase in cell size was observed, particularly among cells morphologically identified as podocytes based on their characteristic location in the visceral layer of the glomerular capsule. These alterations were accompanied by extensive mesangial expansion, compatible with focal segmental glomerulosclerotic alterations. In addition, focal obliteration of glomerular capillaries was evident, along with glomeruloparietal synechiae, reflecting a structural compromise of the glomerulus compared with the other groups (Figure 4G,H).

### 3.4. Stereological Findings

**Figure 5:**
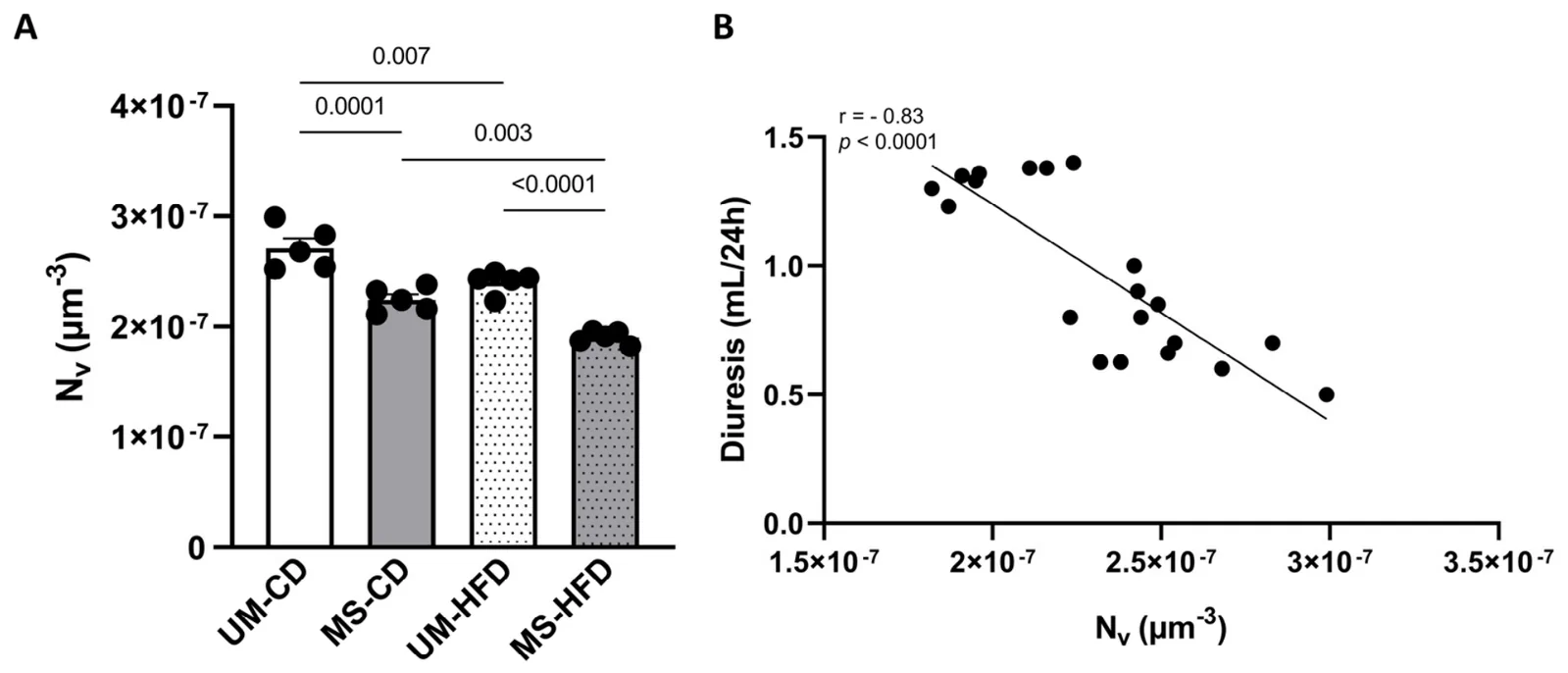
Effect of early maternal separation stress and high-fat diet on the number of glomeruli per μm^3^ and its relationship with urinary function. (**A**). Glomerular N_v_. (**B**). Inversely proportional correlation between glomerular N_v_ and diuresis. Values are presented as mean ± SEM; dots represent individual animals (n = 5 per group). Significant differences are indicated with a *p* < 0.05. UM-CD, unmanipulated with control diet; UM-HFD, unmanipulated with high-fat diet; MS-CD, maternal separation with control diet; MS-HFD, maternal separation with high-fat diet; Nv, numerical density.

The UM-CD group had the highest number of glomeruli per μm^3^, representing the reference point among the experimental groups. Compared with this group, moderate and comparable decreases in glomerular numerical density (N_v_) were observed in both the MS-CD (−17.3% vs. UM-CD) and UM-HFD (−11.4% vs. UM-CD) groups. Given the coexistence of both factors, the MS-HFD group showed the most marked decrease (−15.2% vs. MS-CD; −20.8% vs. UM-HFD), reflecting a progressive decline in glomerular N_v_ under experimental conditions. Consistent with these findings, the two-way statistical analysis revealed that both manipulation (59.80% of the total variance) and diet (26.85% of the total variance) had a significant effect on glomerular N_v_. Conversely, for this parameter, no significant interaction was observed between the two factors, indicating that both factors contribute independently to variation in the evaluated parameter. These results were consistent with the multiple comparisons analysis, in which significant differences were observed between the UM-CD group and the MS-CD (*p* = 0.0001) and UM-HFD (*p* = 0.007) groups, as well as between the MS-HFD group and the MS-CD (*p* = 0.003) and UM-HFD (*p* < 0.0001) groups (Figure 5A). Taken together, these results showed that exposure to a single experimental factor, either MS or HFD, was sufficient to induce a reduction in the number of glomeruli per μm^3^, while their combination in the MS-HFD group produced a cumulative, but not synergistic, effect.

Considering that both Nv and diuresis showed consistent variations between the experimental groups, the association between these two parameters was evaluated, identifying, using Spearman’s coefficient, a strong negative correlation between both variables (r = –0.83; *p* < 0.0001), a finding that indicates that, as the number of glomeruli per μm^3^ decreases, there is greater urinary excretion (Figure 5B).

**Figure 6:**
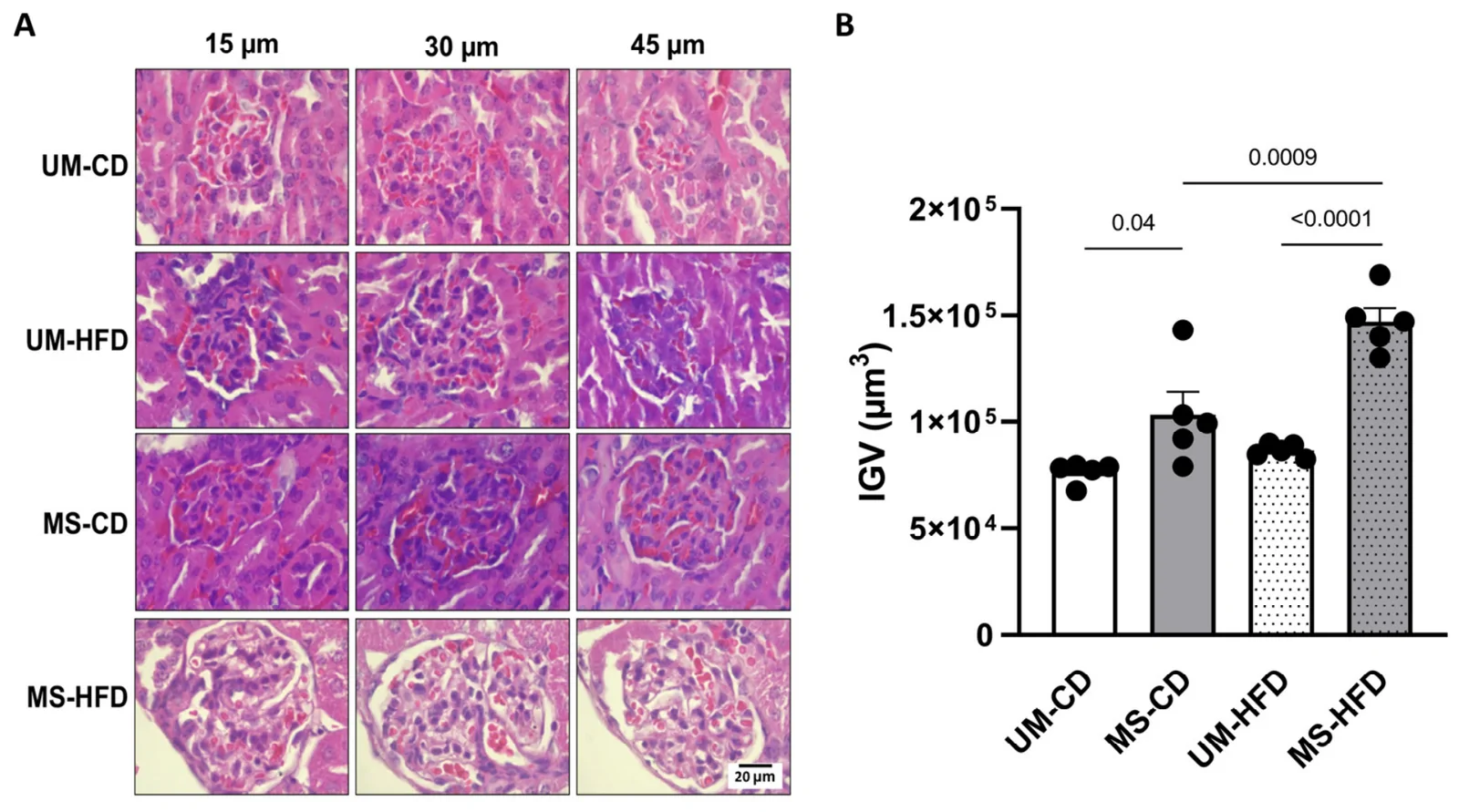
Histological and quantitative evaluation of IGV in mice from the UM-CD, UM-HFD, MS-CD, and MS-HFD groups. (**A**). Representative histological sections of glomeruli obtained at different section depths (15, 30, and 45 µm), illustrating the variations in the glomerular profile according to the depth of section. H&E staining. (**B**). IGV in the four experimental groups. Data are presented as mean ± SEM. *p* < 0.05; dots represent individual animals (n = 5 per group). IGV, individual glomerular volume; UM-CD, unmanipulated with control diet; UM-HFD, unmanipulated with high-fat diet; MS-CD, maternal separation with control diet; MS-HFD, maternal separation with high-fat diet; H&E, hematoxylin and eosin.

For individual glomerular volume (IGV), the UM-CD group had the lowest values, constituting the reference point among the experimental groups. Compared with this group, a moderate increase was observed in MS-CD (+35.5% vs. UM-CD), whereas the increase was milder in UM-HFD (+13.3% vs. UM-CD). The coexistence of both factors in the MS-HFD group resulted in the most marked increase in IGV (+42.0% vs. MS-CD; +69.8% vs. UM-HFD), which showed a progressive pattern of glomerular hypertrophy under the experimental conditions (Figure 6A). In accordance with these findings, the two-way analysis of variance revealed significant effects of manipulation (53.23% of total variance) and diet (20.35% of total variance) on IGV, as well as a significant interaction between both factors (7.89%), indicating that the cooperation of MS and HFD enhances the increase in IGV. Post hoc analyses using Tukey confirmed significant differences between UM-CD versus MS-CD (*p* = 0.040), between MS-CD versus MS-HFD (*p* = 0.0009), and between UM-HFD versus MS-HFD (*p* < 0.0001), while no significant differences were observed between UM-CD and UM-HFD (*p* = 0.68) (Figure 6B). Taken together, these results indicate that exposure to MS alone was sufficient to significantly increase IGV, whereas HFD alone did not produce a significant increase in IGV. However, combined exposure to MS and HFD was associated with a further increase in IGV compared with MS-CD. However, the combination of both factors generated a synergistic effect that exceeded that observed with either factor alone, demonstrating partial potentiation of glomerular hypertrophy.

**Figure 7:**
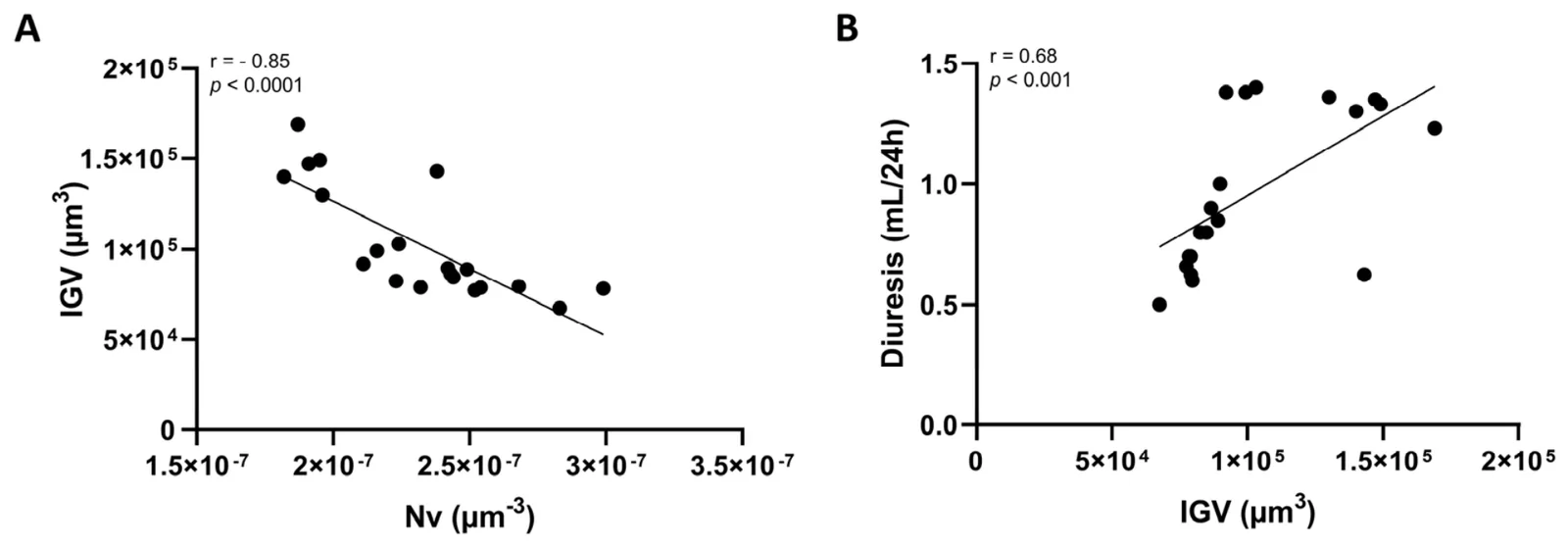
Correlations between glomerular and functional parameters. (**A**). Inversely proportional correlation between glomerular Nv and IGV. (**B**). Directly proportional correlation between IGV and diuresis. Nv, number density; IGV, individual glomerular volume.

Using Pearson’s coefficient, a very strong and significant inversely proportional relationship was identified (r = −0.85; *p* < 0.0001) between glomerular Nv and IGV, suggesting that as the number of glomeruli per μm^3^ decreases, IGV increases (Figure 7A). Similarly, a moderate and significant positive correlation was found between IGV and diuresis (r = 0.68; *p* < 0.001), indicating that a higher IGV is associated with an increase in urinary excretion (Figure 7B). Table 2 details the findings of the two-way ANOVA.

## 4. Discussion

The results of this study provide evidence that both MS and post-weaning exposure to an HFD induce glomerular morphofunctional alterations in C57BL/6 male mice. However, factor analyses showed that both factors did not act in the same way on the evaluated parameters. While diuresis and Nv showed cumulative effects without significant interaction between manipulation and diet, renal mass and IGV showed significant interaction, suggesting that the coexistence of early stress and metabolic overload partially enhances some renal remodeling processes. Taken together, these findings indicate that both factors act through partially distinct, yet convergent, mechanisms that increase susceptibility to glomerular damage.

In general, both MS and HFD promoted changes consistent with early renal remodeling. HFD induced a significant increase in renal mass, while MS showed a predominant effect on diuresis. Previous studies have shown that HFD induces low-grade chronic inflammation, cellular infiltration, and tissue remodeling in metabolically active organs, including the kidney [29,30]. Furthermore, early stress models have been associated with hemodynamic alterations and persistent neuroendocrine responses that modify renal function in later stages of life [31].

Regarding renal mass, the significant interaction observed suggests that early stress partially modulates the response induced by HFD. This behavior could be related to inflammatory and adaptive mechanisms associated with metabolic overload. Several studies have shown that HFD induces renal lipid accumulation, inflammation, and progressive tissue remodeling [16,29]. Furthermore, early exposure to stress has been associated with persistent neuroendocrine alterations that modify cell proliferation and renal tissue organization [13,32]. Furthermore, the metabolic changes observed in this same experimental model provide a relevant context for interpreting the alterations in renal mass identified in the present study. In complementary studies conducted by our research group using the same animals, HFD produced significant increases in body mass, of approximately 40.6% in UM-HFD compared with UM-CD and 35.7% in MS-HFD compared with MS-CD [33], while alterations in the lipid profile were also observed, particularly an increase in total cholesterol associated with both HFD and MS [34]. These changes could contribute to the increased renal mass observed in HFD-exposed groups, as the metabolic burden associated with increased adiposity and altered lipid homeostasis may favor renal remodeling and lipid accumulation, mechanisms that have been linked to structural and functional renal alterations [35]. In this regard, the magnitude of this response may be partially conditioned by MS-induced programming, whereas the greater metabolic demand associated with HFD may increase renal workload and promote adaptive structural changes, including glomerular hypertrophy and tissue remodeling [14,16,29]. If these adaptive responses are sustained over time, they may acquire maladaptive characteristics, favoring the progression of glomerular injury and contributing to alterations in renal function [14,36,37]. Nevertheless, renal mass should be understood as an overall morphometric parameter rather than a direct measure of renal hypertrophy. In the present study, hypertrophy was specifically assessed through IGV, providing a more direct measure of glomerular enlargement. Therefore, changes in renal mass may reflect an overall process of structural remodeling but, by themselves, do not constitute evidence of functional impairment.

Diuresis was the functional parameter most influenced by MS. The observed pattern was consistent with the cumulative effects of both factors, suggesting that MS and HFD contribute to increased urine volume through different mechanisms. In this context, manipulation emerged as the main determinant of the observed variability, while diet represented an additional factor of lesser magnitude.

Early stress models for MS have been shown to induce persistent sympathetic activation, neurogenic hypertension, and renal hemodynamic alterations in adulthood [31]. Although increased renal sympathetic activity generally promotes sodium and water reabsorption, sustained increases in blood pressure may activate pressure-natriuresis and pressure-diuresis mechanisms through increased renal perfusion pressure, which reduces tubular sodium and water reabsorption and consequently favors their excretion [38,39]. This mechanism could contribute to the increased diuresis observed in the groups subjected to MS. Furthermore, HFD is an experimental model widely associated with obesity, insulin resistance, and metabolic alterations that affect renal function [40,41]. In this context, HFD could further contribute to increased diuresis through osmotic and hemodynamic mechanisms related to altered glucose handling and renal tubular load [42].

Histological analysis revealed a structural progression of glomerular damage consistent with the experimental conditions. While the UM-CD group had preserved glomerular architecture, the experimental groups exhibited progressive alterations characterized by reduced capsular space, mesangial expansion, focal capillary obliteration, and glomeruloparietal synechiae. In particular, the MS-HFD group presented alterations, including podocyte hypertrophy, extensive mesangial expansion, and changes compatible with focal segmental glomerulosclerosis. These findings are consistent with alterations described in glomerulopathies associated with obesity and diabetes, where lipotoxicity, inflammation, and hemodynamic overload favor progressive glomerular remodeling [14,41,42]. In this context, the histological alterations observed become particularly relevant when considered alongside the metabolic environment previously characterized in this same cohort [33,34]. Greater adiposity and altered lipid homeostasis may favor lipotoxicity, inflammation, and hemodynamic stress, thereby promoting glomerular remodeling and injury [35]. This interpretation is particularly relevant to the greater severity of the alterations observed in MS-HFD, in which mesangial expansion, focal capillary obliteration, glomeruloparietal synechiae, and podocyte hypertrophy may reflect the convergence of renal susceptibility previously conditioned by MS with subsequent exposure to an adverse metabolic environment.

The decrease in glomerular Nv observed in the experimental groups suggests that both MS and HFD contribute to the deterioration of glomerular structural integrity. The pattern observed for this parameter was consistent with additive effects of both factors, indicating that early stress and metabolic overload would act through distinct, though convergent, pathophysiological mechanisms contributing to the reduction in glomerular Nv.

MS represented the factor with the largest effect size on glomerular Nv, supporting the idea that early stress is an important modulator of renal programming. Considering that in mice a significant portion of nephrogenesis occurs during the first few postnatal days and culminates around PND5 [5,43], exposure to stress during this critical window could interfere with normal renal development processes.

Several studies have shown that neonatal stress induces sustained activation of the hypothalamic–pituitary–adrenal axis and increases circulating glucocorticoids [10,12]. Experimentally, excessive exposure to glucocorticoids during renal development has been associated with alterations in nephrogenesis and reduction in nephron capacity [2]. In addition, mechanisms related to alterations in regulatory pathways of renal development, such as GDNF/RET, could contribute to compromising ureteral branching and the proper formation of nephrons [44,45]. Thus, this background suggests that MS could favor a lower glomerular endowment by altering early renal development.

Additionally, the reduction in glomerular Nv could also be related to mechanisms after nephrogenesis, associated with progressive injury and functional loss of previously formed glomeruli. In this context, experimental studies have shown that early stress induces persistent inflammatory activation in the adult kidney [13], while other models of chronic stress have shown decreased glomerular number and renal morphometric alterations [46]. These findings are consistent with our histological findings, particularly the mesangial expansion, focal capillary obliteration, and changes compatible with glomerulosclerosis observed in the MS-treated groups.

Regarding the effect of diet, given that HFD exposure occurred in a post-nephrogenic period, it is unlikely that the decrease in glomerular Nv is related to direct alterations in nephron formation. In contrast, the findings suggest the involvement of mechanisms associated with progressive glomerular injury. Previous studies have shown that HFD induces renal lipid infiltration, inflammation, mesangial expansion, and early glomerular alterations [16,29]. Likewise, obesity associated glomerulopathy is characterized by glomerular hypertrophy, podocyte injury, and progression to glomerulosclerosis [14]. Taken together, these mechanisms could contribute to the reduction in glomerular Nv observed in the groups exposed to HFD.

In accordance with these structural findings, the strong negative correlation between glomerular Nv and diuresis suggests a close relationship between a lower number of glomeruli per μm^3^ and renal functional alterations. The reduction in the number of glomeruli favors compensatory hyperfiltration responses in the remaining nephrons, increasing the tubular load and promoting initial renal functional modifications [15,47]. Thus, the alterations observed in diuresis could represent early manifestations of glomerulotubular uncoupling associated with progressive renal remodeling.

In addition to the reduction in glomerular Nv, our results showed a significant increase in IGV in the experimental groups, particularly in animals simultaneously exposed to MS and HFD. In contrast to the glomerular Nv, the IGV showed a significant interaction between both factors, suggesting partial potentiation of the observed glomerular hypertrophy.

In this context, glomerular hypertrophy is one of the main adaptive responses observed following a reduction in the number of nephrons, increasing filtration by individual glomeruli [6,48]. MS represented the main determinant of the increase in IGV, even in the absence of dietary intervention, suggesting that early stress could induce persistent alterations in renal hemodynamics and glomerular susceptibility. Several studies have shown that neonatal stress models generate sustained neuroendocrine responses, sympathetic activation, and hemodynamic alterations capable of modifying renal function in later stages of life [12,31].

On the other hand, although HFD had a smaller effect than MS on IGV, its combination with early stress produced the most severe alterations. Previous studies have shown that HFD induces renal lipotoxicity, mesangial expansion, inflammation and podocyte injury, processes that favor progressive glomerular remodeling [14,16,29]. In this context, the coexistence of a lower programmed glomerular endowment early on, along with post-weaning metabolic overload, could create a scenario particularly susceptible to the development of compensatory hypertrophy.

The strong inverse correlation observed between glomerular Nv and IGV supports this interpretation, indicating a decrease in glomerular number is directly associated with a compensatory increase in glomerular size. This pattern is consistent with experimental models of nephron loss, where sustained hyperfiltration promotes glomerular hypertrophy and progression to glomerulosclerosis [48,49]. Furthermore, the positive correlation between IGV and diuresis suggests that glomerular structural alterations are associated with initial renal functional modifications, probably related to increased filtration surface and progressive tubular overload.

From a pathophysiological perspective, our results support the hypothesis that a lower number of nephrons per μm^3^ would favor compensatory mechanisms of hyperfiltration and glomerular hypertrophy, progressively increasing susceptibility to chronic kidney damage [6,15,48]. In this context, early exposure to stress could serve as an initial programming event, with post-weaning exposure to HFD further amplifying renal metabolic and hemodynamic overload, accelerating the progression of glomerular damage.

In particular, the MS-HFD group presented the most severe structural alterations, suggesting that the combination of neonatal stress and postnatal metabolic challenge generates a more vulnerable renal phenotype. These findings are consistent with the concept of “double aggression” described in developmental programming, where adverse early exposures increase susceptibility to later metabolic risk factors [2]. Taken together, our findings suggest that MS and HFD may act through partially distinct but convergent mechanisms on the kidney. MS may increase renal susceptibility through developmental programming and neuroendocrine and hemodynamic alterations [2,12,31,50], whereas HFD subsequently imposes a metabolic burden associated with lipid accumulation, inflammation, and glomerular remodeling [14,16,29]. The coexistence of both factors may limit renal adaptive capacity and promote compensatory responses such as glomerular hypertrophy, contributing to greater glomerular remodeling and injury. This interpretation is consistent with our findings, in which MS and HFD showed additive effects on glomerular number, whereas the significant interaction observed for IGV suggests that early exposure to MS may modify the renal response to the subsequent metabolic challenge.

From a translational perspective, these results become relevant considering the global increase in early adverse experiences, obesity, and metabolic diseases during childhood and adolescence. In this sense, our findings suggest that early psychosocial and nutritional factors could interact in a complementary way on renal susceptibility, promoting structural alterations and initial renal functional modifications that could precede the clinical development of CKD.

However, this study has some limitations. The total volume of the renal cortex was not estimated using the Cavalieri method, and therefore the present stereological analysis was limited to the estimation of glomerular numerical density rather than the total number of glomeruli within the kidney. The lack of molecular, inflammatory, oxidative stress, and hemodynamic analyses prevents us from precisely identifying the pathways underlying the observed glomerular alterations. Furthermore, metabolic parameters and direct markers of renal hyperfiltration were not evaluated. In addition, renal functional assessment was primarily based on urine output, and additional biochemical and urinary markers of renal function were not evaluated. The different weaning ages between the MS and UM groups represent an inherent limitation of the model and should be considered when interpreting the observed effects. Nevertheless, the integration of histological, quantitative, and functional analyses allowed for a consistent characterization of the renal phenotype induced by MS and HFD. Furthermore, based on background from similar experimental models [50], this study provides novel evidence of the interaction between early stress and postnatal metabolic overload in the programming of glomerular damage.

## 5. Conclusions

MS and post-weaning exposure to a high-fat diet (HFD) induced renal structural and functional alterations in male C57BL/6 mice. MS was associated with alterations in diuresis and glomerular numerical density (Nv), while HFD primarily influenced renal mass. A significant interaction between MS and HFD was observed for individual glomerular volume (IGV), indicating that the combined exposure influenced glomerular size through an interaction between the two factors. Furthermore, the coexistence of MS and HFD produced the most pronounced glomerular histological alterations, accompanied by lower Nv and greater glomerular hypertrophy. Consequently, these findings suggest that early stress is associated with alterations in glomerular numerical density and that these changes are accompanied by compensatory remodeling responses that become more evident in the context of post-weaning metabolic overload.

## Figures and Tables

**Table 1:** The diets were administered according to the experimental group: control diet (CD) and high-fat diet (HFD).

Ingredients	CD	HFD
Casein (g/kg) (>85% protein)	200.0	230.0
L-cystine (g/kg)	3.0	3.0
Cornstarch (g/kg)	529.486	299.472
Sucrose (g/kg)	100.0	100.0
Soybean oil (g/kg)	70.0	70.0
Lard (g/kg)	-	200.0
Fiber (g/kg)	50.0	50.0
Vitamin mixture (g/kg)	10.0	10.0
Mineral mixture (g/kg)	35.0	35.0
Choline bitartrate (g/kg)	2.5	2.5
Antioxidant (g/kg)	0.014	0.028
Total (g)	1000.0	1000.0
Energy (kcal/g)	3.95	4.95
Carbohydrate (% Energy)	64.0	32.0
Protein (% Energy)	19.0	19.0
Lipid (% Energy)	17.0	49.0

**Table 2:** Detailed results of the two-way ANOVA on the morphofunctional parameters of the kidney of C57BL/6 mice.

Parameters	Manipulation		Diet		Interaction	
	% Total Variation	*p*-Value	% Total Variation	*p*-Value	% Total Variation	*p*-Value
Renal mass (g)	7.00	NS	**33.10**	0.002	20.2	0.01
Diuresis (mL/24 h)	**49.00**	0.0003	13.54	0.03	0.002	NS
N_v_ glomerular (µm^−3^)	**59.80**	<0.0001	26.85	<0.0001	0.06	NS
IGV (µm^3^)	**53.23**	<0.0001	20.35	0.0007	7.89	0.02

The factor with the greatest influence on the total variance is shown in bold for each parameter. When a factor or interaction influence on a parameter does not reach significance, it is described as NS. Nv, number density; IGV, individual glomerular volume; NS, not significant.

## Data Availability

The raw data supporting the conclusions of this article will be made available by the authors, without undue reservation.

## Abbreviations

The following abbreviations are used in this manuscript:

CD	Control diet
CKD	Chronic kidney disease
HFD	High-fat diet
IGV	Individual glomerular volume
MS	Maternal separation
MS-CD	Maternal separation group with control diet
MS-HFD	Maternal separation group with a high-fat diet
N_V_	Numerical density
UM	Unmanipulated
UM-CD	Unmanipulated group with control diet
UM-HFD	Unmanipulated group with a high-fat diet
PND	Postnatal day
SEM	Standard error of the mean
